# Unconventional secretion factor GRASP55 is increased by pharmacological unfolded protein response inducers in neurons

**DOI:** 10.1038/s41598-018-38146-6

**Published:** 2019-02-07

**Authors:** Anna Maria van Ziel, Pablo Largo-Barrientos, Kimberly Wolzak, Matthijs Verhage, Wiep Scheper

**Affiliations:** 1 Department of Functional Genomics, Center for Neurogenomics and Cognitive Research, Vrije Universiteit (VU), Amsterdam, The Netherlands; 2 Clinical Genetics, Amsterdam UMC, location VUmc, Amsterdam, The Netherlands; 3 Alzheimer Center, Amsterdam UMC, location VUmc, Amsterdam, The Netherlands

## Abstract

Accumulation of misfolded proteins in the endoplasmic reticulum (ER), defined as ER stress, results in activation of the unfolded protein response (UPR). UPR activation is commonly observed in neurodegenerative diseases. ER stress can trigger unconventional secretion mediated by Golgi reassembly and stacking proteins (GRASP) relocalization in cell lines. Here we study the regulation of GRASP55 by the UPR upon pharmacological induction of ER stress in primary mouse neurons. We demonstrate that UPR activation induces mRNA and protein expression of GRASP55, but not GRASP65, in cortical neurons. UPR activation does not result in relocalization of GRASP55. UPR-induced GRASP55 expression is reduced by inhibition of the PERK pathway of the UPR and abolished by inhibition of the endonuclease activity of the UPR transducer IRE1. Expression of the IRE1 target XBP1s in the absence of ER stress is not sufficient to increase GRASP55 expression. Knockdown of GRASP55 affects neither induction nor recovery of the UPR. We conclude that the UPR regulates the unconventional secretion factor GRASP55 via a mechanism that requires the IRE1 and the PERK pathway of the UPR in neurons.

## Introduction

Since neurons are non-proliferative and secretory cells, protein homeostasis or proteostasis is of great importance and hence tightly regulated. The endoplasmic reticulum (ER) is a vital organelle for protein synthesis, folding and posttranslational modifications of proteins destined for the secretory pathway. Disturbed ER proteostasis caused by an accumulation of misfolded proteins is defined as ER stress and triggers a homeostatic control mechanism called the unfolded protein response (UPR). ER stress activates the UPR by inducing the dissociation of the chaperone binding immunoglobulin protein (BiP; also known as glucose-regulated protein 78), from the three transmembrane ER stress sensors; protein kinase R (PKR)-like ER kinase (PERK), inositol requiring enzyme 1 (IRE1) and activating transcription factor 6 (ATF6) (reviewed in^1^). Activation of the UPR aims to restore proteostasis after which the UPR is switched off. Upon ER stress the three UPR pathways are employed to increase expression of chaperones, augment protein folding capacity, transiently block protein synthesis and enhance protein degradation^1^. UPR-mediated regulation involves a complex network of transcriptional and translational regulation of which cell-type specific aspects are an intricate feature that is not fully elucidated (see e.g.^2–4^).

In neurodegenerative diseases, including Alzheimer’s disease (AD), proteostasis is severely disturbed, demonstrated by massive accumulation of aggregated proteins that are the key pathological hallmarks. Not surprisingly, UPR activation is a common feature of neurodegenerative diseases (reviewed in^5^). For example, our previous work shows that the UPR is activated in neurons at an early stage in the pathology of AD and Parkinson’s disease^6,7^. In neurodegenerative diseases, UPR activation is considered to be chronic and contribute to the neurodegenerative process, confirmed by studies in animal models^8–11^. Targeting of UPR pathways has come into view for therapeutic intervention (reviewed in^12,13^). Therefore, it is of great importance to study the consequences of UPR activation in neurons.

Recently, unconventional protein secretion was reported as a downstream effect of ER stress^14–16^. Proteins following the conventional secretory pathway enter the ER after which they pass through the Golgi to their final destination, often the plasma membrane or extracellular space^17^. However, some reach these final destinations when ER-Golgi trafficking is blocked^15,16,18–21^. This has led to the identification of alternative secretory pathways that bypass the Golgi, collectively called unconventional secretion^22–24^. Unconventional secretion is typically triggered by cellular stress (reviewed in^25^). It has been hypothesized to function as a compensatory mechanism for dysfunctional protein quality control^26^, an alternative secretory route if conventional secretion is impaired^15,16^ and to mediate stress-induced danger signaling^18,27^. In neuronal cells, unconventional secretory routes are employed by key proteins involved in neurodegenerative diseases and typically induced by cellular stress^20,28–35^.

Accumulating evidence indicates that the Golgi reassembly and stacking proteins (GRASPs) are key players in a conserved stress-induced alternative secretory pathway that bypasses the Golgi^16,18,21,36–39^. Mammalian cells have two GRASP proteins, GRASP65 and GRASP55. Both are located at the cytoplasmic side of the Golgi membrane and act as membrane tethers^40,41^. They interact with golgins GM130 (cis-Golgi)^42,43^ and Golgin-45 (medial-trans-Golgi) respectively^44,45^ and are involved in the stacking of Golgi cisternae^46^. The N-terminal half of these proteins is largely conserved across species and includes the GRASP domain containing two PDZ (Post synaptic density protein 95, Drosophila disc large tumor suppressor and Zonula occludens-1 protein) domains which allow GRASP to tether membranes and interact with and localize other proteins^47,48^. The PDZ domain-mediated properties of GRASP are speculated to either enable the tethering of vesicular and plasma membranes or recruit specific cargos^16,49,50^. In a model of unconventional secretion, trafficking of the ER-retained mutant cystic fibrosis transmembrane conductance regulator (CFTR) via the unconventional secretory pathway, can be triggered by ER stress-inducing treatments and GRASP55 overexpression in mammalian cell lines^16^. This involves phosphorylation and a strong relocalization of GRASP55 from the Golgi to the ER^16,50^.

The effects of ER stress and UPR activation on the unconventional secretion machinery have not been investigated in neurons. Given the chronic neuronal UPR activation in neurodegenerative diseases and its potential as target for therapeutic intervention, it is imperative to elucidate the consequences of UPR activation in the disease-relevant cell type. Therefore, we investigated the regulation of GRASP by the UPR in primary mouse cortical neurons. Our data show that in contrast to cell lines, UPR activation does not induce GRASP55 relocalization in neurons, but increases GRASP55 levels in an IRE1- and PERK-dependent manner.

## Results

### GRASP55 expression is induced by the ER stressor TM in cortical neurons and astrocytes

**Figure 1:**
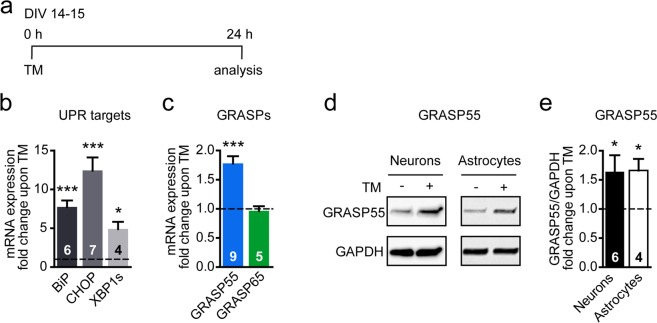
TM induces UPR and GRASP55 expression in cortical neurons and astrocytes. (**a**) Timeline of the experiment performed with TM in primary cortical mouse neurons at DIV14–15. (**b**,**c**) Primary mouse neurons were treated for 24 h with DMSO (vehicle) or TM (5 µg/mL) (as shown in (**a**)) before qPCR analysis is performed, mRNA expression of UPR target genes BiP, CHOP and XBP1s (**b**) and of GRASP55 and GRASP65 (**c**) is shown. Statistical differences of *n* independent experiments (*n* is shown in bars) were measured by the one-sample t-test (two-tailed) compared to baseline (DMSO, set to 1) of each target gene. (**d**,**e**) GRASP55 protein levels were measured using Western Blot analysis after DMSO or TM (5 µg/mL) in mouse primary neurons and astrocytes. GAPDH was used as a reference gene. Representative blots of both cell models are shown in (**d**). Uncropped blots are shown in Fig. S3. Quantification of multiple blots is shown in (**e**). Statistical differences were measured by the one-sample t-test (two-tailed) compared to baseline (DMSO, set to 1) for *n* independent experiments (*n* is shown in bars).

Both GRASP55 overexpression and ER stress-inducing treatments can trigger trafficking via the unconventional secretory pathway in cell lines^16^. Therefore, we hypothesize that ER stress induces GRASP55 expression in primary neurons. To induce ER stress, neurons were treated with tunicamycin (TM), an inhibitor of N-linked glycosylation^51,52^ for 24 hours and with TM and thapsigargin (TG), a non-competitive inhibitor of the sarcoplasmic/ER Ca^2+^ ATPase (SERCA) pump^53^ for 6 hours. This results in an activated transcriptional UPR, confirmed by the increased expression of the downstream UPR target genes BiP (Figs 1b, S1b), C/EBP homologous protein (CHOP) (Fig. 1b) and the spliced variant of X-Box binding Protein-1 (XBP1s) (Fig. 1b). No significant decrease in neuronal number is observed indicating that this TM and TG treatment protocol does not induce pronounced apoptosis (Fig. S2a,b and d,e). A 6 hour-treatment with TM and TG showed a small upward trend in GRASP55 mRNA expression, but no significant increase (Fig. S1c). However, a 24 hour-TM treatment did significantly induce mRNA expression of GRASP55 (1.8 ± 0.4 fold; Fig. 1c), whereas GRASP65 expression was not affected (neither after 6 hours (Fig. S1c) nor 24 hours (Fig. 1c)). Protein levels of GRASP55 were also increased in both primary neurons (1.6 ± 0.3 fold) and astrocytes (1.7 ± 0.2 fold) upon 24 hours of TM (Figs 1d,e, S3). A knockdown experiment confirmed that the antibody used, specifically detects GRASP55 (Fig. S4a). These data show that TM induces GRASP55, but not GRASP65 expression in neurons and astrocytes.

### GRASP55 does not relocalize upon ER stress-inducing treatments in cortical neurons

**Figure 2:**
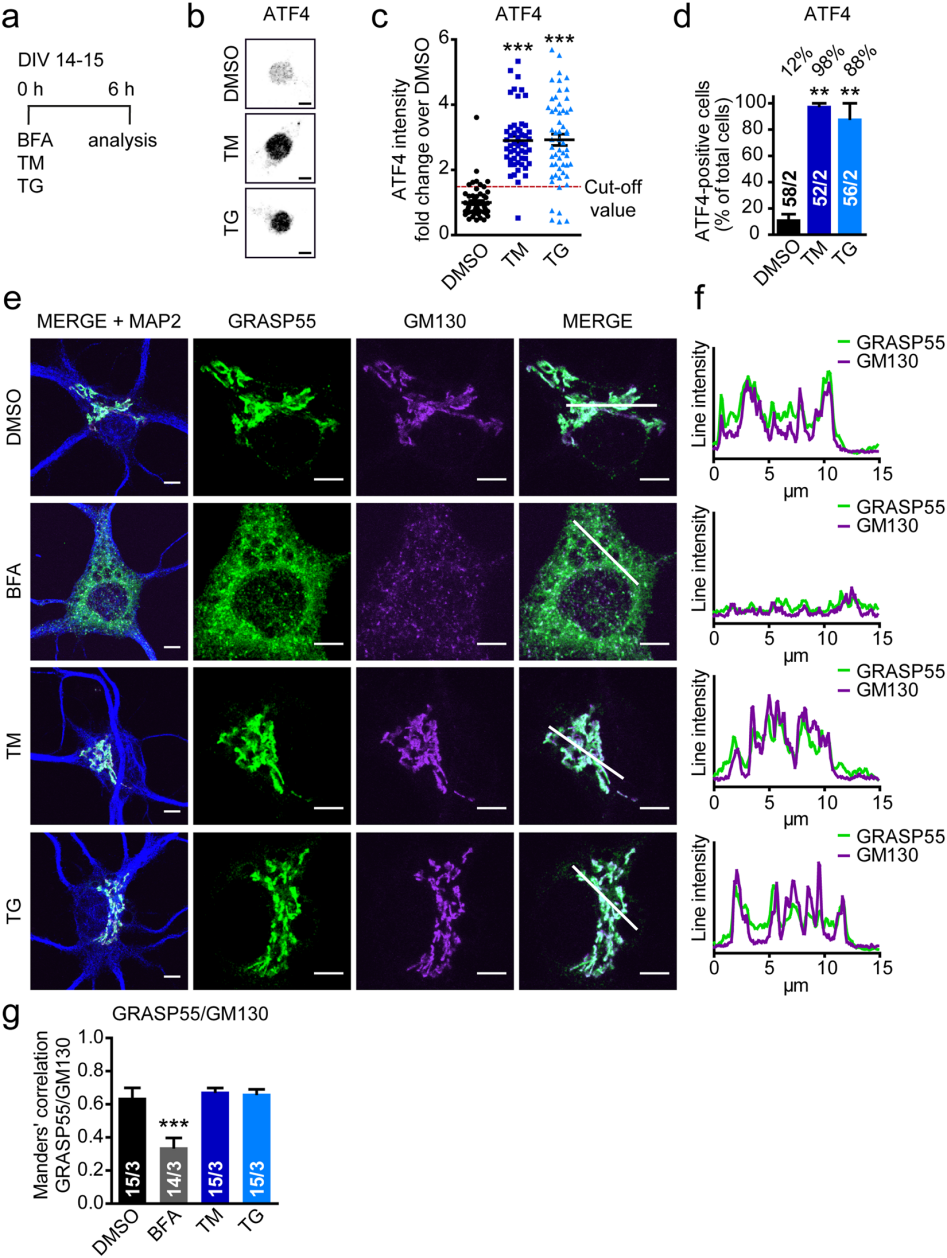
Treatments inducing the UPR do not result in GRASP55 relocalization in cortical neurons. (**a**) Timeline of the experiment with BFA, TM and TG in primary cortical mouse neurons (DIV14–15). (**b**–**d**) Neurons were treated with DMSO (vehicle), TM (5 µg/mL) or TG (1 µM) (as shown in (**a**)) and stained for UPR activation marker ATF4. Typical examples are shown in (**b**) scale bar is 5 µm. Quantification of fluorescence intensity of ATF4 in the nucleus is shown in (**c**), every dot represents a single cell. Data was normalized to mean DMSO per experiment and shown as fold change over mean DMSO. The red dotted line represents the cut-off value (mean + SD of DMSO). All cells with a fluorescence intensity above this value were considered ATF4-positive. The percentage of total cells considered ATF4-positive per experiment is represented in (**d**) for both TM and TG. The number of analyzed cells *n* in 2 independent experiments (*n*/2) is shown in bars. Significant differences in (**c,d**) were tested via one-way ANOVA followed by post hoc Tukey’s multiple comparison test compared to DMSO. (**e**) Representative images of neurons treated as in (**a**–**d**) with DMSO (vehicle), BFA (10 µg/mL), TM (5 µg/mL) or TG (1 µM) and stained for dendritic marker (MAP2, blue), Golgi marker (GM130, magenta) and GRASP55 (green). Overview images of complete cell body (MERGE + MAP2) and more detailed images of Golgi and GRASP55 stainings are shown. Scale bar in overview and enlarged images is 5 µm. (**f**) Line tracings of fluorescence intensities to visualize colocalization of GM130 (magenta) and GRASP55 (green) are shown. Location of line tracings is represented as a white line in MERGE images. (**g**) Colocalization of GRASP55 with the Golgi (GM130) was evaluated with the Manders’ correlation coefficient in neurons treated as in (**e**). Statistical differences of *n* analyzed cells in 3 independent experiments (*n*/3 is shown in bars) compared to DMSO were measured by the Kruskal-Wallis test followed by Dunn’s multiple comparison test.

GRASP55 relocalization from the Golgi to the ER upon ER stress-inducing treatments has been implicated in the regulation of unconventional membrane trafficking in transformed cells^50^. To study whether a similar mechanism applies to neurons, the subcellular localization of endogenous GRASP55 in HeLa cells and primary cortical mouse neurons was studied upon ER stress-inducing treatments. A 24 hour-UPR-inducing treatment did not induce relocalization in neurons or astrocytes (Fig. S5). However, in HeLa cells an acute treatment of 1 hour was sufficient to induce GRASP55 relocalization^54^. Therefore, we addressed relocalization in both HeLa cells and neurons using a short treatment protocol (1 or 6 hours in HeLa cells (Fig. S6) and 6 hours in neurons (Fig. 2)). We have previously shown that a 6 hour-treatment with TM induces the UPR in HeLa cells^55^, 1 hour is insufficient to elicit the transcriptional UPR response (not shown). In neurons, UPR activation after 6 hours TM or TG treatment was confirmed on the whole pool of cells by analyzing BiP mRNA expression and by single cell analysis of nuclear staining intensity of activating transcription factor 4 (ATF4)^56^. BiP mRNA expression is increased upon TM and TG treatment (Fig. S1b). ATF4 accumulates in the nucleus in cells with an active UPR (Fig. 2b–d) and 98% and 88% of total cells were ATF4-positive upon TM and TG treatments respectively (Fig. 2d). These treatment paradigms did not induce neuronal death (Fig. S2a,d,e).

Neurons and HeLa cells were treated with brefeldin A (BFA) in order to validate the immunostaining and colocalization analysis. BFA induces collapse of Golgi transmembrane proteins into the ER thereby disrupting Golgi structure thus relocalizing peripheral Golgi proteins like GRASP55 to the cytoplasm^57,58^.

GRASP55 localization was assessed by immunostaining for GRASP55 and colocalization analysis was performed with the Golgi marker GM130. In the vehicle control condition (DMSO), GRASP55 largely colocalized with the Golgi marker GM130 in both HeLa cells (Fig. S6) and neurons (Fig. 2e–g). Upon treatment with BFA, the expected fragmentation of GM130 was observed in both HeLa cells and neurons. This was accompanied by a dispersed pattern of GRASP55 staining throughout the cytoplasm. Colocalization analysis showed a significant decrease in the fraction of GRASP55 overlapping with GM130 in both cell types (Figs 2g and S6c). In HeLa cells, TG did induce a significant decrease in colocalization as was reported previously. Because induction for 1 hour is insufficient to induce a full UPR, we also included a 6 hour treatment. TG treatment also induced a more fragmented Golgi morphology represented by GM130 immunostaining in HeLa cells both after 1 and 6 hours (Fig. S6a,b). TM treatment, however, induced no change in GRASP55 localization. In neurons, no change in GRASP55 localization was observed upon TM or TG treatment. Line intensity plots of the fluorescent signal of GRASP55 and GM130 showed largely similar peaks for DMSO, TM and TG treatments, while BFA treatment produced an overall lower signal in neurons (Fig. 2f). These data indicate that although TG triggers relocalization of GRASP55 in HeLa cells, ER stress induction in general does not induce subcellular relocalization of GRASP55 in neither neurons nor HeLa cells.

### TM-induced GRASP55 expression requires IRE1 activity

**Figure 3:**
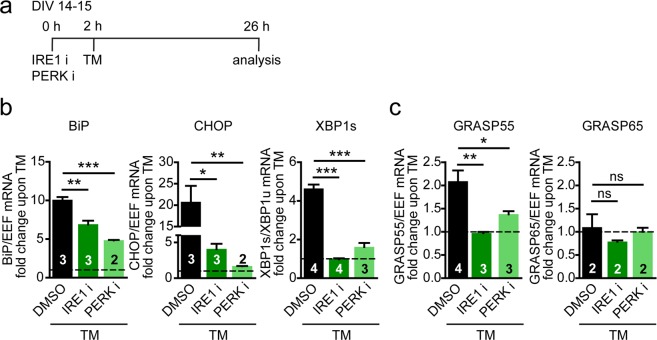
TM-induced GRASP55 expression is inhibited by IRE1 and PERK inhibitors. (**a**) Timeline of experiment with the IRE1 inhibitor (IRE1 i) and the PERK inhibitor (PERK i). (**b**,**c**) Primary cortical neurons were pre-treated for 2 h with DMSO (vehicle), IRE1 inhibitor (50 µM) (4µ8C, inhibitor of RNase activity of IRE1) or the PERK inhibitor GSK2606414 (5 µM), followed by a 24 h incubation with either DMSO (Fig. S7) or TM (5 µg/ml) (**b**,**c**). mRNA expression levels were analyzed by qPCR of UPR target genes (**b**), GRASP55 and GRASP65 (**c**). Data are represented as fold change over respective control conditions. Significant differences of *n* independent experiments (*n* is shown in bars) were measured by one-way ANOVA followed by Tukey’s multiple comparison test, all conditions were compared to DMSO + TM.

To follow up on the observation that TM induces GRASP55 expression, we investigated whether this required a specific UPR signaling pathway in neurons. Since both IRE1^16^ and PERK activity^14^ have been implicated in ER stress-induced unconventional secretion in cell lines, neurons were treated with inhibitors of IRE1 (4µ8C), which specifically blocks the RNase activity of IRE1^59^, or PERK (GSK2606414)^60^. First, the efficacy of the inhibitors in our assay was assessed (Fig. 3a,b). Expression of BiP, CHOP and XBP1s mRNA increased upon TM treatment. The IRE1 inhibitor completely abolished XBP1s expression, a selective target of IRE1. CHOP and BiP levels were also decreased. PERK inhibitor treatment blocked expression of its downstream target CHOP and also reduced BiP and XBP1s expression.

Interestingly, the IRE1 inhibitor prevented TM-induced GRASP55 expression (Fig. 3c). PERK inhibitor treatment also inhibited GRASP55 induction, but to a lesser extent than the IRE1 inhibitor. GRASP65 was not affected by any of the UPR inhibitors (Fig. 3c). Inhibition of IRE1 or PERK activity in the absence of ER stress had no effect on GRASP55 or GRASP65 expression (Fig. S7).

### Active XBP1s is not sufficient to induce GRASP55 expression

**Figure 4:**
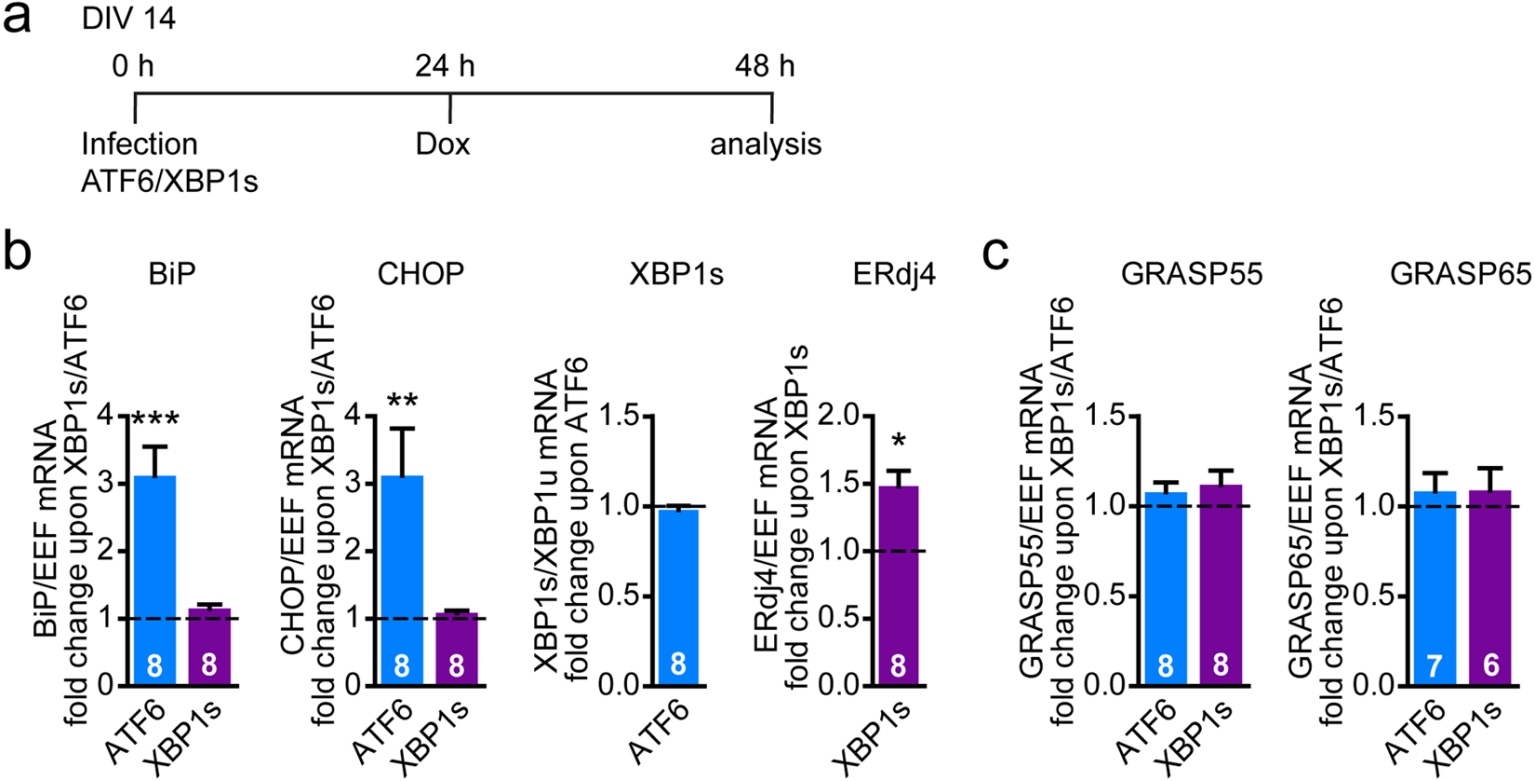
Active XBP1s is not sufficient to induce GRASP55 expression. (**a**) Timeline of experiment with overexpression of Dox-inducible active transcription factors ATF6 and XBP1s. (**b**,**c**) Lentiviral transduction was used to express active transcription factors XBP1s and ATF6 in primary cortical neurons. XBP1s and ATF6 should only be expressed upon Dox addition (TetON construct). Timeline as represented in (**a**). mRNA expression levels were analyzed by qPCR of UPR target genes (**b**), GRASP55 and GRASP65 (**c**) upon Dox addition (1 µg/ml). Data are shown as fold change difference over the expression of a control construct with a similar backbone (TetON) in the presence of Dox (baseline, set to 1). Significant differences of *n* independent experiments (*n* is shown in bars) were tested via one-way ANOVA followed by post hoc Dunnett’s multiple comparison test compared to baseline.

To further address the involvement of the IRE1 pathway in GRASP55-induced expression in more detail, we developed a novel tool to specifically activate signaling downstream of IRE1: Doxycycline (Dox)-induced overexpression of the active form of the transcription factor XBP1. As a control for UPR pathway-selective transactivation we employed expression of active ATF6 in the same expression system. Without Dox addition, no UPR targets were induced by either XBP1s or ATF6 (Fig. S8a). To confirm the activity of the overexpressed XBP1s and ATF6, the induction of some of their respective established UPR target genes was determined^61–64^ (Fig. 4b). Upon Dox-induced ATF6 overexpression, BiP and CHOP were induced ~3-fold, whereas XBP1s expression was unaffected. Dox-induced XBP1s expression did not affect BiP and CHOP levels. The activity of overexpressed XBP1s was confirmed by the increased expression of its downstream target gene ERdj4^63,64^. These data confirm the Dox-regulated activity and specificity of the overexpressed active transcription factors XBP1s and ATF6 in neurons.

Overexpression of active XBP1s was employed to investigate whether the transcriptional response of the IRE1 pathway was sufficient to induce GRASP55 expression. Neither XBP1s nor ATF6 overexpression induced a significant change in GRASP55 mRNA levels upon Dox treatment (Figs 4c, S8b without Dox addition). GRASP65 mRNA levels were also not affected by XBP1s or ATF6 overexpression (Fig. 4c). These data indicate that overexpression of active XBP1s in the absence of ER stress and without an active PERK pathway is not sufficient to induce GRASP55 expression.

### GRASP55 does not affect UPR recovery in neurons

**Figure 5:**
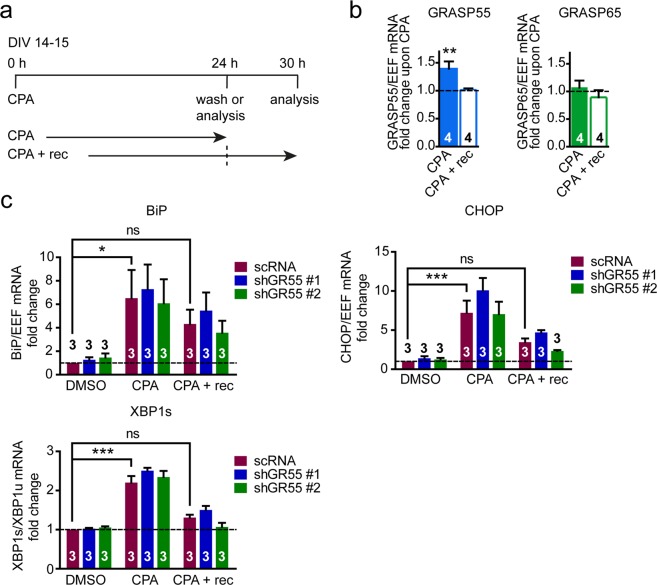
GRASP55 is reversibly induced by CPA and not required for UPR recovery in cortical neurons. (**a**) Timeline of the experiment in primary mouse neurons at DIV14–15 treated with DMSO (vehicle) or CPA (100 µM), analyzed before (CPA/DMSO) or after total medium change and a recovery period of 6 h (CPA + rec). (**b**) Cortical neurons were treated as in (**a**) and mRNA expression of GRASP55 and GRASP65 is analysed by qPCR. Statistical differences were assessed by a one-way ANOVA followed by Dunnett’s multiple comparison test compared to baseline (DMSO). (**c**) Mouse primary cortical neurons were infected with either scrambled shRNA (scRNA), or with shRNA’s targetting GRASP55 (shGR55 #1 or shGR55 #2) for 4 days before treatment, as in (**a**,**b**). mRNA expression of UPR target genes BiP, CHOP and XBP1s was analysed after CPA or CPA + rec treatments. Bar graphs represent mean ± SEM of 3 independent experiments. Statistical differences were assessed by a two-way ANOVA, no significant effect was observed between the scRNA and shRNA groups. Significance was detected between the different CPA treatments (DMSO, CPA, CPA + rec), therefore a Bonferroni’s post-hoc test was performed to compare the different CPA treatments to DMSO in the scRNA condition.

To strengthen the data demonstrating GRASP55 is increased upon UPR activation and to further investigate the regulation of GRASP55 by the UPR, an ER stress-inducing agent with a different mode of action than TM was employed, cyclopiazonic acid (CPA). Like TG, CPA is an inhibitor of the SERCA Ca^2+^ pump. However, unlike TG, CPA is a reversible inhibitor, allowing the study of UPR induction and recovery upon wash out which was previously examined in MEF cells^65^. In neurons, 24 hours incubation with CPA did not significantly decrease neuronal number (Fig. S2a,c). Also by this different UPR induction paradigm, GRASP55 expression was induced (Fig. 5a,b). Upon CPA wash out (CPA + rec), GRASP55 levels returned to baseline. GRASP65 expression was unaffected in this paradigm (Fig. 5b). These data are in line with the data in Fig. 1 indicating that the UPR specifically regulates GRASP55 expression.

To investigate whether GRASP55 is functionally involved in UPR recovery, the CPA recovery paradigm was employed in neurons infected with two independent lentiviral GRASP55 shRNAs or a control shRNA (scRNA). GRASP55 shRNA expression led to a strong reduction in GRASP55 mRNA and protein levels (reduction in GRASP55 protein: shRNA #1 ~76%, shRNA #2 ~82%; Fig. S4a–c). In the scRNA condition, BiP, CHOP and XBP1s expression were increased upon CPA treatment and this was reversed upon CPA wash out (Fig. 5c). Expression of the UPR targets was not affected by either of the shRNAs in the absence of CPA (DMSO; Fig. 5c). Importantly, GRASP55 knockdown neither affected UPR target expression in the induction phase of the treatment (CPA) nor in the recovery phase (CPA + rec). This demonstrates that GRASP55 does not affect UPR induction and is not required for UPR recovery.

## Discussion

The UPR is activated in neurodegenerative diseases and is a potential target for therapeutic intervention. Therefore, it is pivotal to study the consequences of UPR activation in neurons. Our data indicate that activation of the UPR via two independent pharmacological ER stress inducers (TM and CPA), increases the expression of GRASP55 -but not GRASP65- in neurons. The TM-induced increase is inhibited by inhibitors of the IRE1 and PERK pathway of the UPR.

As was previously reported^50,54^ we show that in HeLa cells the ER stress inducer TG triggers relocalization of GRASP55. TG treatment alters Golgi morphology, indicated by Golgi marker GM130, making a direct conclusion difficult. Importantly, TM treatment did not result in GRASP55 relocalization. Therefore, it is unlikely that general ER stress induces GRASP55 relocalization in HeLa cells. A more plausible explanation could be that altered Golgi function and morphology induce the changes in GRASP55 subcellular localization, since ER-to-Golgi blockade has the same effect on GRASP55^50^. In neurons, our experiments using two independent ER stress paradigms and two different timepoints, provided no evidence for UPR-induced relocalization of GRASP55, while blocking ER-to-Golgi transport using BFA did show clear relocalization (Figs 2e–g, S5). Although we cannot exclude that a limited fraction of GRASP55 is redistributed to vesicular carriers or membranes^18,38,66^, we conclude that the UPR does not regulate the subcellular localization of GRASP55 in neurons.

In mammalian cell lines, increased levels of GRASP55 are sufficient to induce unconventional trafficking of the ER-retained mutant CFTR^16^. In addition, upregulation of the *Drosophila* (d)GRASP ortholog, was shown to activate unconventional integrin secretion^19,38^. In these model systems cellular stress is a trigger for GRASP55-mediated unconventional secretion: ER stress-inducing treatments lead to unconventional CFTR trafficking in cell lines^16^ and dGRASP is upregulated upon mechanical stress^19^. Since stress-induced increased levels of GRASP55 result in unconventional secretion in cell lines and Drosophila, it would be interesting to test if ER stress-induced upregulation of GRASP55 in primary neurons also leads to unconventional secretion. We were unable to establish a reliable unconventional secretion assay using mutant CFTR in neurons, therefore functional assays await identification of neuronal cargo for GRASP55-mediated unconventional secretion.

Here we show that GRASP55 upregulation is blocked by the IRE1 inhibitor 4μ8C in neurons suggesting IRE1 endonuclease activity is essential for the induction of GRASP55. The involvement of the IRE1 pathway in GRASP55-dependent unconventional secretion was also demonstrated in cell lines, however in contrast to our results this was attributed to an effect of basal IRE1 levels on GRASP55 phosphorylation^16^ whereas our data indicate the involvement of the ER stress-induced IRE1 RNase activity on GRASP55 levels in neurons. Our data show that PERK inhibition results in a partial block of TM-induced GRASP55 expression (Fig. 3c) demonstrating the involvement of both the IRE1 and PERK pathway in UPR-mediated GRASP55 increase. This may be due to the fact that the three UPR pathways are not isolated routes but display extensive crosstalk^67,68^ (Figs 3b, 4b). This is illustrated by the expression of BiP, which is reduced by both inhibitors (Fig. 3b), although to a lesser extent than the more pathway-selective targets. In addition, the PERK inhibitor strongly affects the expression of the direct downstream target of IRE1, XBP1s (Fig. 3b). This is most likely mediated by ATF4, a transcription factor downstream of PERK. ATF4 enhances IRE1 expression, resulting in increased levels of XBP1s^68^. The effect of both inhibitors on IRE1 signaling could underlie the strong correlation between XBP1s and GRASP55 expression upon treatment with either inhibitor (Fig. 3b,c). This suggests IRE1 signaling is key for GRASP55 upregulation. However, overexpression of active XBP1s alone did not induce GRASP55 (Fig. 4c). Although we cannot exclude that the level of XBP1s overexpression was insufficient, this is unlikely, because the established XBP1s target ERdj4 was induced^63,64^ demonstrating transactivation of XBP1s target genes in our model (Fig. 4b). The complete inhibition of ER stress-induced GRASP55 expression by the IRE1 inhibitor may be (partly) mediated via other targets of the IRE1 endonuclease, as part of regulated IRE1-dependent decay^59^ rather than XBP1s splicing. Alternatively, and in line with the data showing PERK inhibition blocks ER stress-mediated GRASP55 increase, isolated XBP1s expression without ER stress and therefore activity in the PERK pathway may not be sufficient to induce GRASP55 expression.

The key function of the UPR is to restore ER proteostasis after which it will switch off. The UPR may induce GRASP55 to activate unconventional secretion as a way to lower ER protein load and maintain or restore proteostasis. However, we show that the knockdown of GRASP55 does not activate the UPR (Fig. 5c). These results are in line with reported data showing that dGRASP deficiency does not induce UPR markers^69^ and a study in HeLa cells where neither GRASP55 knockdown alone nor in combination with GRASP65 knockdown induced the UPR^70^. In addition, the level of UPR activation is unaffected by the absence of GRASP55 in the UPR recovery paradigm, neither in the activation nor the recovery phase (Fig. 5c). These data suggest GRASP55 is not required for maintaining or restoring ER proteostasis *per se*. It has been shown that cells with impaired proteasomal or lysosomal function activate unconventional secretion to remove misfolded proteins and other cargo^26,28^. It will be interesting to address in future studies whether GRASP55 affects UPR recovery in neurons if the canonical ER protein quality control machinery is overwhelmed or not functional, for example in case of intracellular protein aggregation or aging.

In the present study we show that the UPR induces GRASP55 expression -but not subcellular relocalization- in cortical neurons, via a mechanism that requires the IRE1 and the PERK branch of the UPR. Our results suggest GRASP55 is not involved in maintaining or restoring ER proteostasis. Identification of the neuronal unconventional secretome will provide valuable clues regarding the function of unconventional secretion in neurons and its potential regulation by the UPR.

## Materials and Methods

### Animals

Animal experiments are in accordance with institutional and Dutch governmental guidelines and regulations and were approved by the animal ethical committee of the VU University/VU University Medical Center (“Dier ethische commissie (DEC)”; license number: FGA 11-03).

### Primary and HeLa cell cultures

HeLa cells were maintained in culture flasks in Dulbecco’s Modified Eagle Medium (DMEM) with 4.5 g/L Glucose and UltraGlutamine I (Lonza) supplemented with 10% of heat inactivated Fetal Bovine Serum (HI-FBS) (Gibco) and 0.1% Pen-Strep (Gibco) at 37 °C, 5% CO_2_. Cells were counted and seeded at a density of 10 × 10^4^ cells/well in 12 well plates on 18 mm glass coverslips coated with a solution of 0.01% poly-L-ornithin (Sigma-Aldrich) and 2.5 µg/ml laminin (Sigma-Aldrich).

For neuronal cultures, embryos were obtained by caesarean section of wild type mice at embryonic day 18. Astrocytic cultures were prepared from mice at postnatal day 1. Cortical hemispheres were dissected and collected in ice-cold Hanks’ balanced salt solution (Sigma-Aldrich) with 10 mM HEPES (Hanks-HEPES) (Gibco). For neuronal cultures, 0.25% trypsin (Gibco) was added to Hanks-HEPES for digestion and cortices were incubated for 20 min at 37 °C. For astrocytic cultures, digestion was achieved by addition of 3% papain solution (1% of 100 mM CaCl2 (Sigma), 1% of 50 mM EDTA (AppliChem), 196 mg/L of L-Cysteine (Sigma) diluted in DMEM + Glutamax (Gibco) and 150 µL of papain (Worthington)) and incubated for 45 min at 37 °C. After washing, cortices were incubated with inactivating solution (10% HI-FBS) (Gibco), 2.5 g/L of Albumin Bovine Fraction V (Applichem) and 2.5 g/L of Trypsin-Inhibitor type II-O (Sigma)) diluted in DMEM + Glutamax for 15 min at 37 °C. For both cultures, tissue was washed and triturated with a 1 ml and a fire-polished Pasteur pipette. Astrocytes were first cultured in DMEM to 90% confluency in a culture flask before they were counted and plated. For immunocytochemistry, 7.5 × 10^4^ cells/well were plated in 12 well plates on 18 mm glass coverslips. For qPCR and Western blotting, cells were plated in 6 well plates with a density of 3 × 10^5^ cells/well and for the nuclear count assay in black 96 well plates with 1.5 × 10^4^ cells/well. Both glass coverslips (Fisher Emergo) and plates were coated with a solution of 0.01% poly-L-ornithin (Sigma-Aldrich) and 2.5 µg/ml laminin (Sigma-Aldrich). Neurons and astrocytes (when confluent) were grown in neurobasal medium (Gibco) supplemented with 2% B-27 (Gibco), 18 mM HEPES, 0.25% glutamax (Gibco) and 0.1% Pen-Strep (Gibco) at 37 °C, 5% CO_2_.

### Chemicals, plasmids and lentiviral infections

To induce UPR activation, either tunicamycin (TM) (Sigma-Aldrich), thapsigargin (TG) (Sigma-Aldrich) or cyclopiazonic acid (CPA) (Cayman chemical company) was used. Brefeldin A (BFA) (Sigma-Aldrich) was used to induce Golgi collapse. Cells were treated with the IRE1α inhibitor 4µ8C (Tocris) and the PERK inhibitor GSK2606414 (Axon Medchem) to inhibit respective branches of the UPR. Truncated ATF6, spliced XBP1 and control constructs were cloned into the vector pDESTSIN-TRE-Syn-rtTA2 (cloned from a construct received from Dr. P. E. Schätzle), producing a TetON lentiviral vector where expression is controlled by doxycycline (Dox) (Sigma-Aldrich). pLKO.1 plasmids encoding short hairpin (sh)RNAs against mouse GRASP55 (Table 1) and the scrambled control (scRNA) plasmid were obtained from the MISSION shRNA library (Sigma-Aldrich). The deltaCre-GFP construct was a kind gift from P. Kaeser, M.D. Lentiviral particles of pLKO.1, TetON plasmids and delta-Cre-GFP were generated as described before^71^. Concentrations and incubation times of chemicals and lentiviruses are indicated in figure legends.

**Table 1:** Sequence of the shRNAs used for GRASP55 knockdown. TRC numbers refer to numbers in the MISSION shRNA library (Sigma-Aldrich).

shRNA	TRC number	Oligo sequence
shGR55#1	TRCN0000077520	CCGG **GCTATGGTTATTTGCACCGAA** CTCGAG **TTCGGTGCAAATAACCATAGC** TTTTTG
shGR55#2	TRCN0000077521	CCGG **CCCTGTCATGACTACTGCAAA** CTCGAG **TTTGCAGTAGTCATGACAGGG** TTTTTG

### Immunocytochemistry

Experiments in HeLa cells were performed 1day after seeding followed by fixation. Neurons and astrocytes were fixed after 14–15 days *in vitro* (DIV14–15). All cell types were fixed in 2% formaldehyde (Electron Microscopy Sciences) and incubated for 10 minutes (min) followed by incubation in 4% formaldehyde for 20 min at room temperature (RT). After washing in phosphate-buffered saline (PBS, pH 7.4), cells were permeabilized in 0.5% Triton X-100 (Fisher Chemical) in PBS for 5 min and blocked using 0.1% TritonX-100 and 2% normal goat serum for 30 min. Primary and secondary antibodies were diluted in blocking buffer. Primary antibody incubation was performed overnight at 4 °C. Primary antibodies and dilutions used are; monoclonal rabbit anti-ATF4 D4B8 (1:200; Cell Signaling, #11815), polyclonal rabbit anti-GRASP55 (1:100; Atlas Antibodies, HPA03275), monoclonal mouse anti-GM130 (1:1000; BD Transduction Laboratories, 610822) and polyclonal chicken anti-MAP2 (1:500; Abcam, ab5392). Negative stainings (secondary antibody only incubation) were included. After 3 washes in PBS, coverslips were incubated with Alexa Fluor conjugated secondary antibodies (1:1000; Invitrogen) for 1–2 hours (h) at RT. After 3 washes in PBS (second wash contained 5 µg/ml DAPI (Thermo Scientific)), coverslips were embedded in Mowiol (Sigma-Aldrich).

### Image acquisition and analysis

GRASP55-stained cells were imaged on a Zeiss LSM 510 confocal laser-scanning microscope with a 63x oil immersion objective (NA 1.4) and 3x zoom using LSM510 software, cells were randomly selected by GM130 staining. ATF4-stained neurons were imaged with a NIKON Ti-Eclipse microscope using galvano scanning mode and a 60x oil-immersion objective, cells were randomly selected by MAP2 staining. Z-stacks were acquired with 1 µm interval with 5 slices (ATF4 and GRASP55 in HeLa cells), 6 slices (GRASP55 in astrocytes) and 7 slices (GRASP55 in neurons). Analyses were performed with ImageJ software and z-stacks were collapsed to maximal projection. ATF4 immunofluorescence was measured in a region of interest drawn around the nucleus. Cells were considered ATF4-positive when the nuclear intensity was higher than the cut-off value (mean + 1 SD of DMSO control cells). GRASP55 colocalization analysis was performed using plugin JACoP. For Manders’ coefficient the threshold was determined manually per experiment and kept constant for the analysis of cells throughout all the different conditions.

### Western blotting

Neurons were lysed in freshly prepared ice-cold PBS with 1% Triton X-100 (Fisher Chemical), protease inhibitors (Roche) and phosphatase inhibitor cocktails (Roche). Cells were incubated for 15 min on ice before they were scraped, collected and cleared by centrifugation for 20 min at 14000 rpm and 4 °C. Total protein concentration in the supernatants was determined by a Bradford protein assay (Bio-Rad). Equal amount of lysates, containing SDS loading buffer, were incubated for 5 min at 98 °C prior to protein separation on 4–15% polyacrylamide precast gels (Bio-Rad). Proteins were transferred to nitrocellulose membranes (Bio-Rad) by using the Trans-Blot Turbo Transfer system (Bio-Rad). Membranes were briefly washed in tris-buffered saline with Tween 20 (TBS-T) (Sigma-Aldrich) before 1 h incubation in blocking buffer (5% milk powder (Merck) in TBS-T). Primary antibodies (polyclonal rabbit anti-GRASP55 (1:500; Atlas Antibodies, HPA03275); monoclonal mouse anti-GAPDH (1:5000; Merck Millipore, MAB374)) were diluted in blocking buffer and membranes were incubated overnight at 4 °C. After washing in TBS-T, membranes were incubated with horseradish peroxidase (HRP)-labeled antibodies (Dako) (1:2000) in blocking buffer for 1 h at RT and again washed in TBS-T. Blots were developed using the Lumi-Light reagent (Roche) for 5 min and visualized on the Odyssey Imaging System (LI-COR). Intensity of protein bands was quantified with Image Studio 2.0 software, using the GAPDH signal to correct for total amount of protein.

### RNA isolation, cDNA synthesis and Real-Time quantitative PCR

Cells were lysed and scraped in TRI Reagent solution (Invitrogen) and lysates were added to Phase Lock Gel Heavy tubes (Quantabio). Organic and aqueous phase separation was initiated by addition of and mixing with chloroform (1:5) (Merck), before centrifugation at 12000 g for 10 min at 4 °C. RNA isolation was performed using the Isolate II RNA minikit (Bioline) including a DNase treatment according to manufacturer’s protocol. The NanoDrop 1000 spectrophotometer (Thermo Scientific) was used to asses RNA concentration, purity and integrity. Synthesis of cDNA was performed using the SensiFAST cDNA Synthesis Kit (Bioline) according to manufacturer’s protocol. Per sample 1 µl cDNA was pipetted in triplicate in a 384-well plate suitable for qPCR (Greiner). Primers and probe (Roche) combinations are provided in Table 2. SensiFAST Probe No-ROX kit (Bioline) or SensiFAST SYBR No-ROX kit (Bioline) for XBP1s/u, were added to the primer-probe mixtures to enable the qPCR reaction. The Advanced Relative Quantification analysis of the LightCycler 480 software was used for analysis.

**Table 2:** Primers and probes used for qPCR. Sequence of the primers and their corresponding probes. Probe numbers refer to numbers in the universal probe library (Roche).

Target gene	Primer sequence 5′-3′	Universal probe/SYBR green
BiP	fw: GCCAACTGTAACAATCAAGGTCT rev: TGACTTCAATCTGGGGAACTC	#15
CHOP	fw: CCACCACACCTGAAAGCAG rev: TCCTCATACCAGGCTTCCA	#33
XBP1s	fw: TCCGCAGCAGGTGCAG rev: CCAACTTGTCCAGAATGCCC	SYBR green
XBP1u	fw: GCAGCACTCAGACTATGTG rev: CCAACTTGTCCAGAATGCCC	SYBR green
ERdj4 (DNAJB9)	fw: TGTGTGTAGTCACTCTTTTGCACT rev: TTGCACATAATAAGGTTACACAGAAA	#71
GRASP55	fw: CATGTGCTGGAAGTGGAATC rev: GCTGAACAGGTCTTCAGACTCA	#3
GRASP65	fw: ACACGTGTGGCATGTGCT rev: GAGCCAACTATGTAGTCTGTGTAAGG	#62
EEF1A1	fw: ACACGTAGATTCCGGCAAGT rev: AGGAGCCCTTTCCCATCTC	#31

### Nuclear count assay

At DIV1 neurons were infected with deltaCre-GFP lentivirus, the deltaCre targets the GFP to the nucleus. Treatments as indicated in figure legend, were performed at DIV14 followed by formaldehyde fixation (as described in the immunocytochemistry section). Neurons were imaged using a cellomics array scan (CellInsight CX7 High-Content Screening (HCS) Platform; ThermoFisher Scientific) with a 10x objective, 15 fields of view per well. Quantification was performed with Columbus 2.5 software (PerkinElmer).

### Statistical analysis

Graphpad Prism 5.0 software was used to perform statistical analysis. The statistical tests used are mentioned in each figure legend. Bar graph values represent mean ± SEM of *n* independent experiments (*n* is shown in bars) unless otherwise stated in figure legend. For qPCR data, mRNA levels of EEF1A1 (EEF) are used as reference gene for BiP, CHOP, GRASP55 and GRASP65. XBP1s values are shown as the ratio of XBP1s over XBP1u. A p value of ≤0.05 was considered statistically significant. *p ≤ 0.05, **p ≤ 0.01, ***p ≤ 0.001, ns indicates not significant.

## Supplementary information

Supplementary figures

## Data Availability

The data generated and analyzed during the current study are included in this published article and its supplementary information files. Datasets are available from the corresponding author on request.
