# Supplementary material for: Unconventional secretion factor GRASP55 is increased by pharmacological unfolded protein response inducers in neurons
Source: Sci Rep. 2019 Feb 7;9:1567. doi: 10.1038/s41598-018-38146-6 (PMC6367349; doi:10.1038/s41598-018-38146-6)
Supplement: Supplementary file 1 — Supplementary figures [file 41598_2018_38146_MOESM1_ESM.pdf]

Unconventional secretion factor GRASP55 is increased by pharmacological unfolded protein response inducers in neurons

Scientific Reports

Anna Maria van Ziel[1,2], Pablo Largo-Barrientos[1] , Kimberly Wolzak[1], Matthijs Verhage[1,2] and Wiep Scheper[1,2,3*]

[1]Department of Functional Genomics, Center for Neurogenomics and Cognitive Research, Vrije Universiteit (VU), Amsterdam, Netherlands.
[2]Clinical Genetics and [3]Alzheimer Center, Amsterdam UMC location VUmc, Amsterdam, Netherlands.

* Correspondence to W. Scheper, w.scheper@vumc.nl, +31-20-5982771

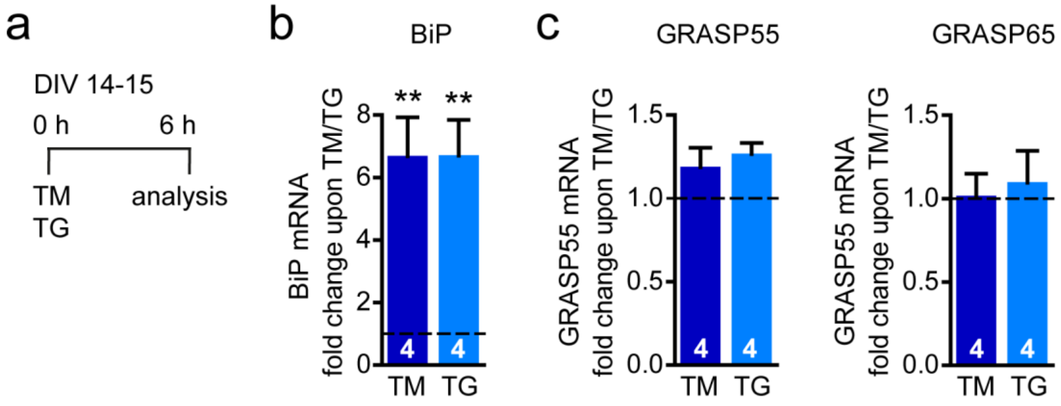

Fig. S1 GRASP55 and GRASP65 mRNA expression is not significantly increased upon 6 h TM or TG

(**a**) Timeline of the experiment performed with TM and TG in primary cortical mouse neurons at DIV14-15. (**b-c**) Primary neurons were treated for 6 h with DMSO (vehicle), TM (5 µg/mL) or TG (1µM) (as shown in (**a**)) before qPCR analysis is performed. mRNA expression of UPR target gene BiP (**b**) and GRASP55 and GRASP65 (**c**) is shown. Significant differences compared to baseline (DMSO, set to 1) in (**b-c**) were tested of *n* independent experiments (*n* is shown in bars) via one-way ANOVA followed by post hoc Tukey's multiple comparison test.

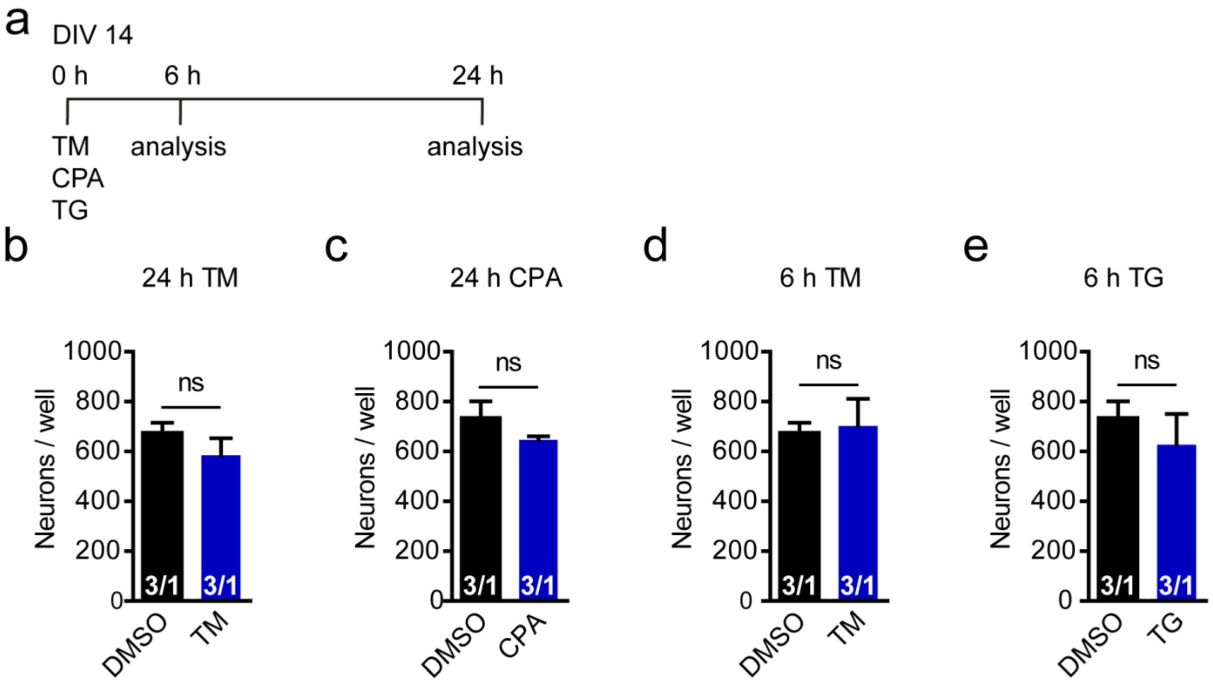

Fig. S2 UPR-inducing treatments do not induce neuronal death in primary cortical neurons

(**a-e**) Primary cortical neurons (DIV 14) were treated with DMSO (vehicle), TM (5 µg/mL), CPA (100 µM), and TG (1 µM) for 24 h (**b-c**) or 6 h (**d-e**) before fixation and automated nuclear count using a cellomics array scan. Data are presented as neurons/well (without normalization; mean ± SD). Statistical differences of *n* analyzed wells in 1 experiment (*n*/1 is shown in bars) compared to DMSO were measured by an unpaired t-test (two-tailed).

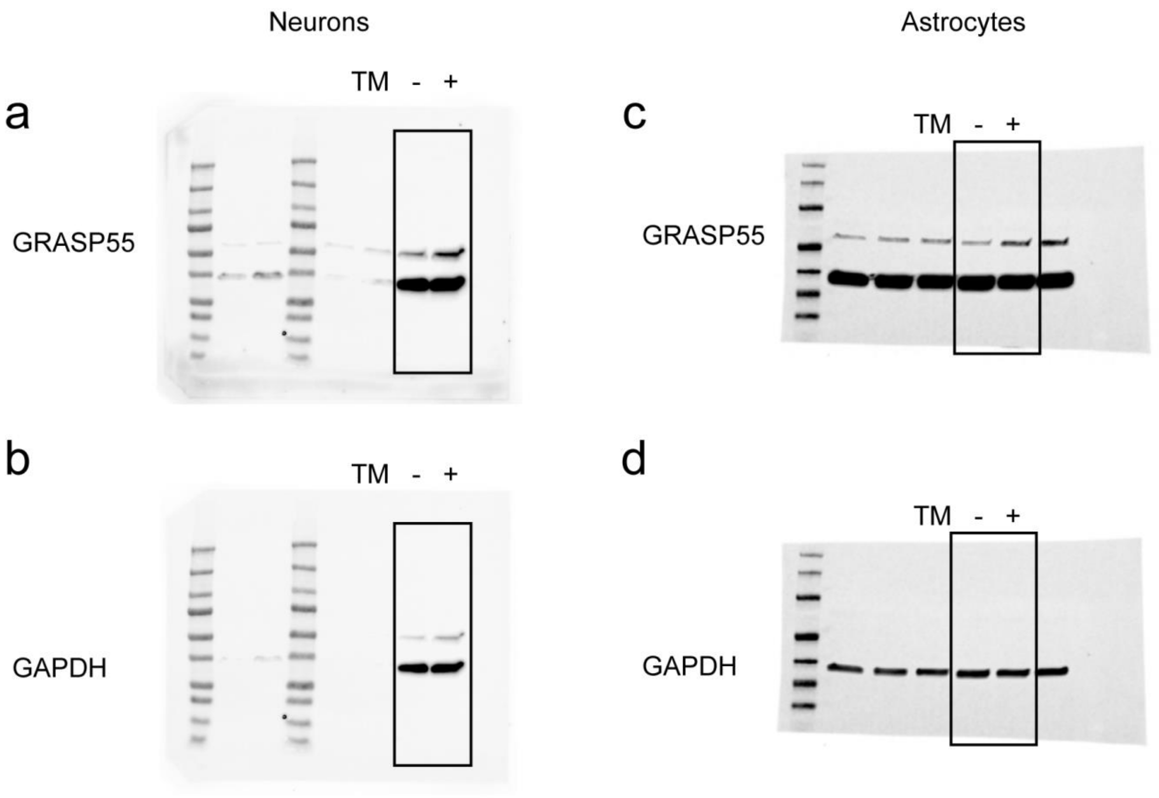

Fig. S3 Uncropped blots showing induced expression of GRASP55 upon TM in cortical neurons and astrocytes

(**a-d**) GRASP55 protein levels were measured using Western Blot analysis after DMSO or TM (5 µg/mL) in mouse primary neurons (**a, b**) and astrocytes (**c, d**) at DIV14-15. Uncropped blots corresponding to blots shown in Fig. 1d of both GRASP55 (**a, c**) and the reference gene GAPDH (**b, d**) with the same exposure time and contrast adjustments. The samples within the rectangles correspond to the samples shown in Fig. 1d.

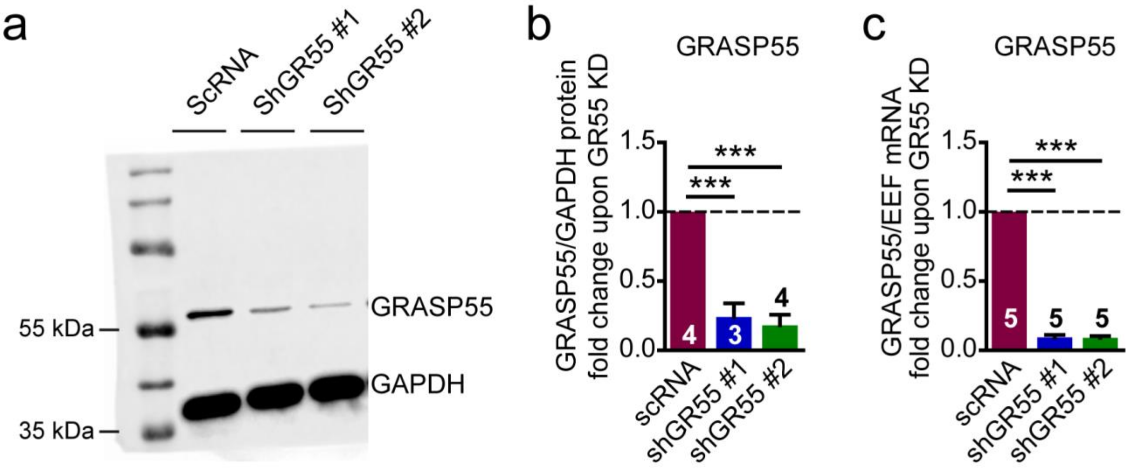

Fig. S4 Validation of GRASP55 knockdown and antibody

(**a**-**b**) Mouse primary cortical neurons were infected with either scrambled shRNA (scRNA), or with shRNA's against GRASP55 (shGR55 #1 or shGR55 #2). GRASP55 protein levels were evaluated after 4 days of infection (DIV14-15) and a representative blot of GRASP55 knockdown is shown (**a**). GAPDH was used as a reference gene. Quantification of 3-4 independent experiments is presented in (**b**), conditions are normalized to scRNA.
(**c**) Mouse neurons were treated as in (**a**) and qPCR analysis was performed after 5 days of infection. mRNA expression of GRASP55 is shown (fold change over scRNA).
Significant differences of *n* independent experiments (*n* is shown in bars) were tested via one-way ANOVA followed by post hoc Dunnett's multiple comparison test compared to scRNA.

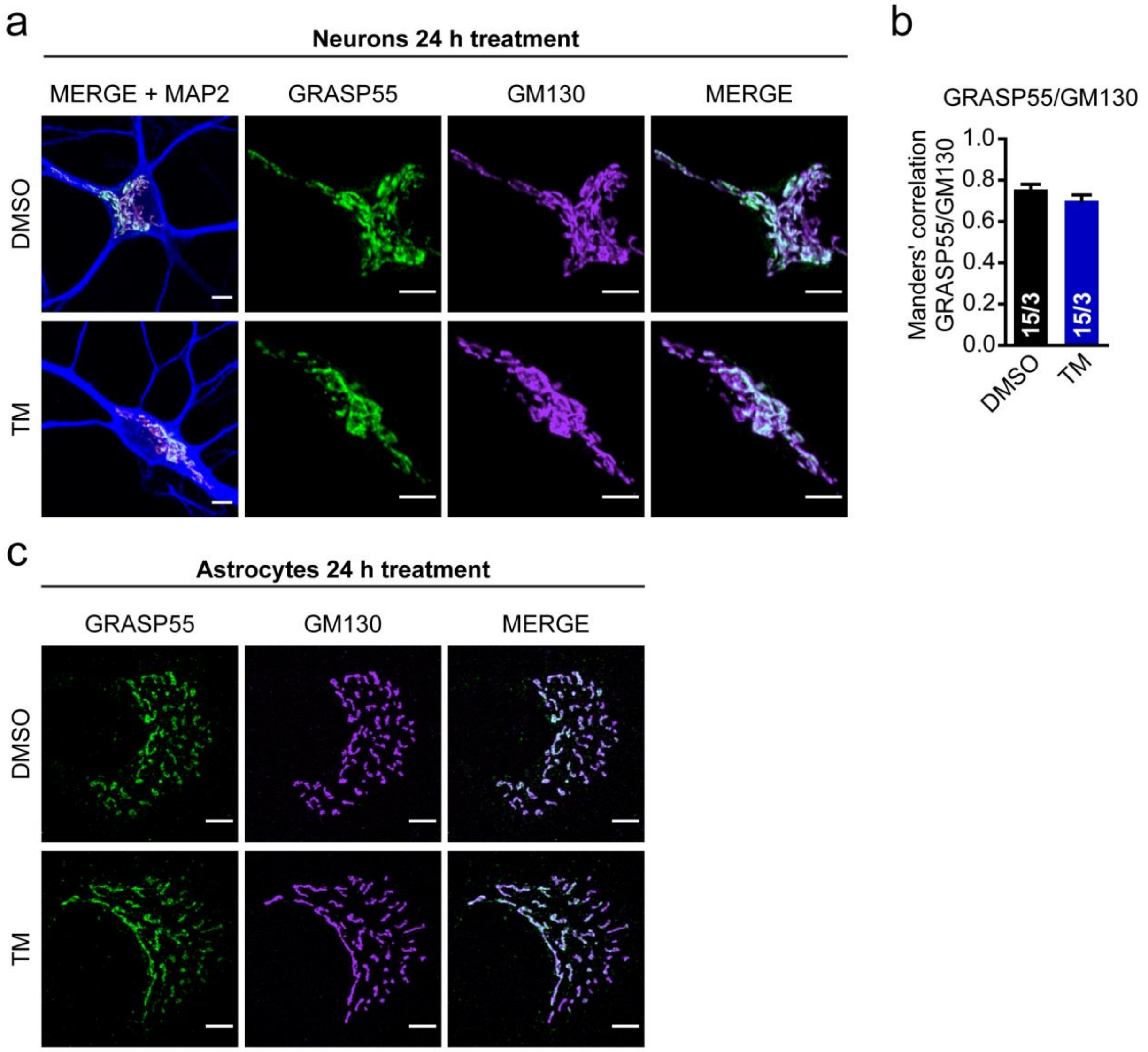

Fig. S5 TM treatment of 24 h does not result in GRASP55 relocalization in neurons and astrocytes

(**a-b**) Neurons were treated with DMSO (vehicle) or TM (5 µg/mL) for 24 h and stained for GRASP55 (green), a Golgi marker (GM130, magenta) and a dendritic marker (MAP2, blue). Representative images (**a**) are shown including overview, zoom and MERGE images. Colocalization of GRASP55 with the Golgi (GM130) was evaluated with the Manders' correlation coefficient for the 24 h treatment in neurons (**b**). No statistical differences were measured compared to DMSO upon analysis of *n* cells in 3 independent experiments (*n*/3 is shown in bars) by an unpaired t-test (two-tailed).

(**c**) Astrocytes were treated as in (**a**) and stained for GRASP55 (green) and a Golgi marker (GM130, magenta). Representative images (n=1) are shown in (**c**). Scale bar is 5 µm in all images.

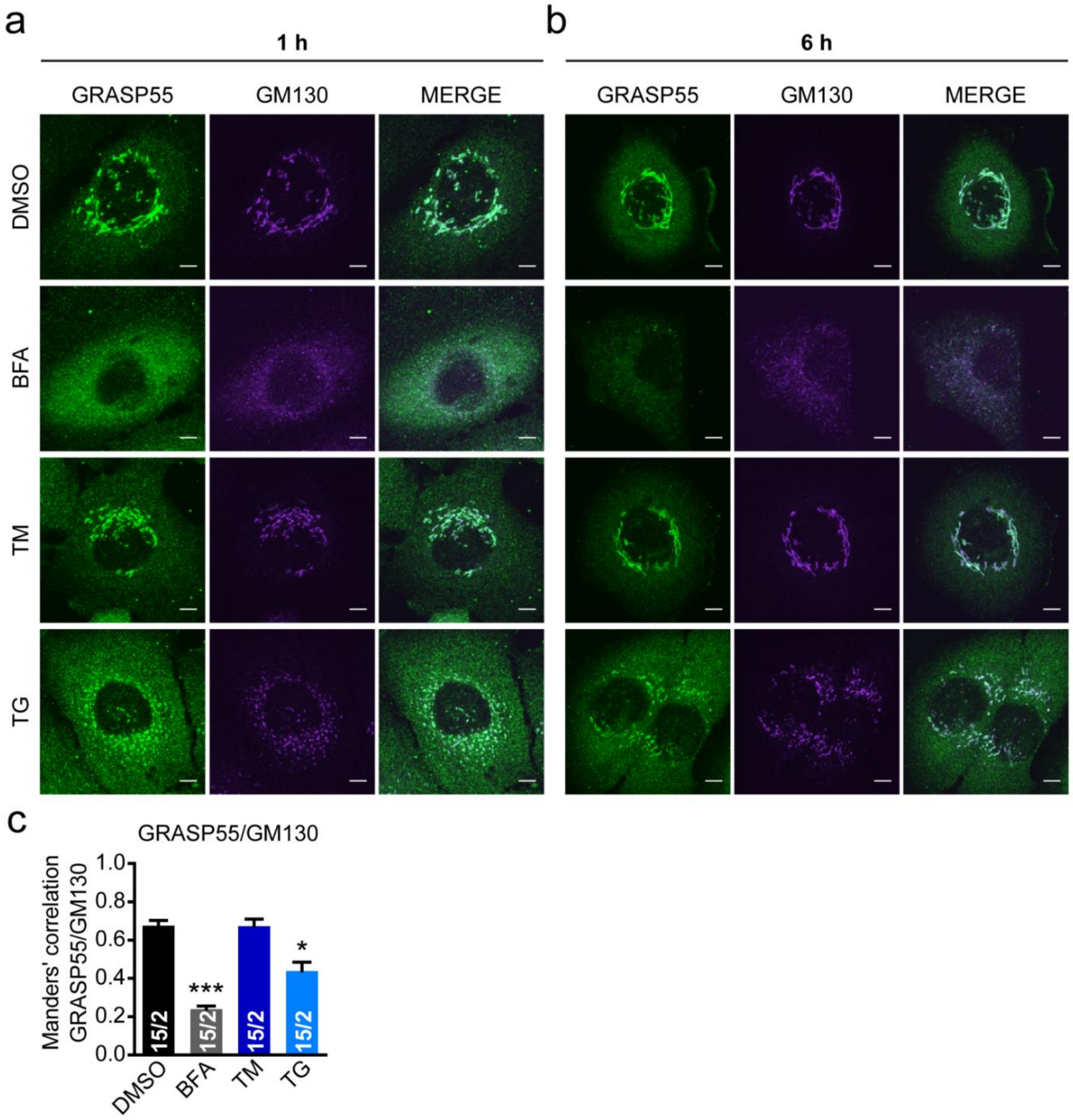

Fig. S6 TG, but not TM, results in GRASP55 relocalization in HeLa cells

(**a-b**) HeLa cells were treated with DMSO (vehicle), BFA (10 µg/mL), TM (5 µg/mL) and TG (5 µM) for 1 h (**a**) or 6 h (**b**). Representative images of HeLa cells stained for Golgi marker (GM130, magenta) and GRASP55 (green) including MERGE images of both GM130 and GRASP55 are shown. Scale bar is 5 µm.

(**c**) Colocalization of GRASP55 with the Golgi (GM130) was evaluated with the Manders' correlation coefficient for the 1 h treatment in HeLa cells. Statistical differences of *n* analyzed cells in 2 independent experiments (*n*/2 is shown in bars) compared to DMSO were measured by the Kruskal-Wallis test followed by Dunn's multiple comparison test.

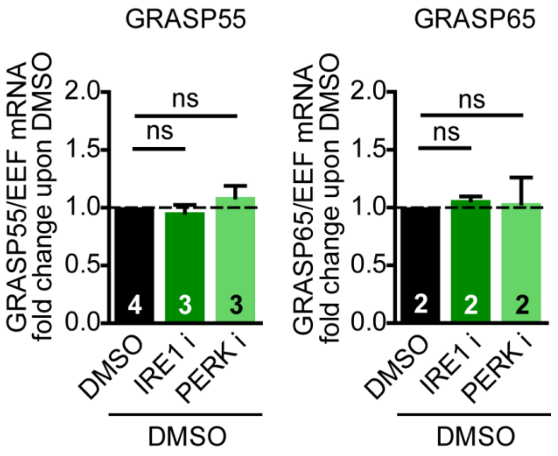

Fig. S7 Inhibition of IRE1 and PERK activity in the absence of ER stress does not affect GRASP55 or GRASP65 expression

Primary cortical neurons were pre-treated for 2 h with DMSO (vehicle), IRE1 inhibitor (IRE i; 4µ8C, inhibitor of RNase activity of IRE1; 50 µM) or the PERK inhibitor GSK2606414 (PERK i; 5 µM), followed by a 24 h incubation with either DMSO (Fig. S2) or TM (Fig. 3c). mRNA expression levels were analyzed by qPCR of GRASP55 and GRASP65 and presented as fold change over DMSO. Significant differences were tested via one-way ANOVA followed by post hoc Tukey’s multiple comparison test, all conditions were compared to DMSO.

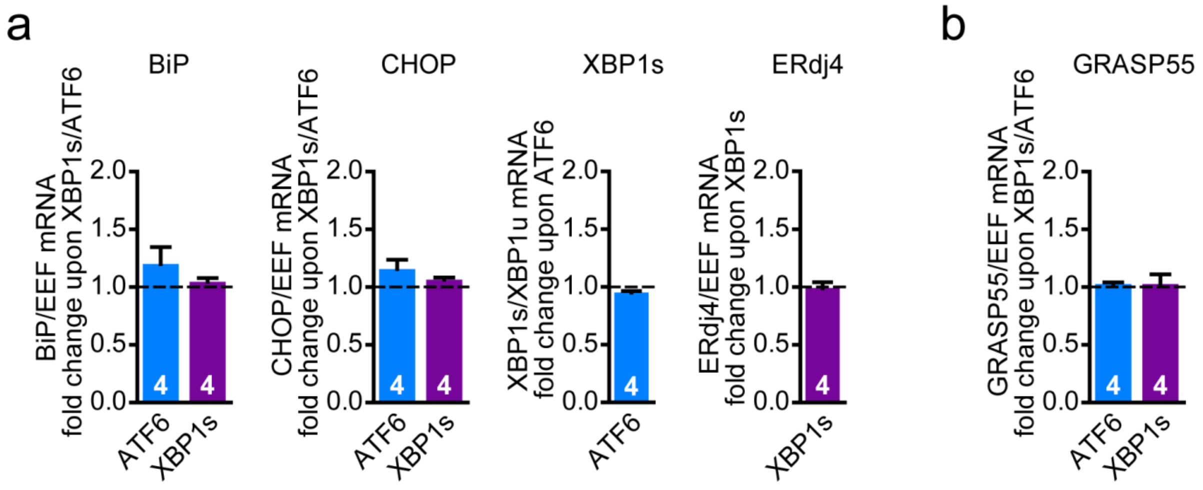

Fig. S8 Validation of inducible active transcription factors ATF6 and XBP1s in the absence of doxycycline (**a-b**) Lentiviral transduction was used to express active transcription factors XBP1s or ATF6 in primary cortical neurons as in Fig. 3a, d-e. mRNA expression levels were analyzed by qPCR of UPR target genes (**a**) and GRASP55 (**b**). Data are shown as fold change difference over the expression of a control construct with a similar backbone (TetON) in the absence of Dox (baseline, set to 1). Significant differences were tested via one-way ANOVA followed by post hoc Dunnett's multiple comparison test compared to baseline.
